# Challenging case of *tinea corporis* and *faciei* in psoriatic patient treated with bimekizumab: The usefulness of mycological screening before biological therapies

**DOI:** 10.1016/j.mmcr.2024.100683

**Published:** 2024-11-16

**Authors:** Terenzio Cosio, Ruslana Gaeta Shumak, Cristiana Borselli, Fabio Artosi, Roberta Gaziano, Elena Campione

**Affiliations:** a Department of Experimental Medicine, University of Rome “Tor Vergata”, 00133, Rome, Italy; b Dermatology Unit, Department of Systems Medicine, University of Rome “Tor Vergata”, 00133, Rome, Italy; c DYNAMYC UR 7380, Faculté de Santé, Université Paris-Est Créteil (UPEC), 94010, Créteil, France

**Keywords:** Bimekizumab, Psoriasis, Interleukin-17, *Tinea corporis*, *Tinea faciei*

## Abstract

Psoriasis is a multifactorial immune-mediated disorder linked to the interleukin (IL)-17 signalling pathway. We present an unusual case of *tinea corporis* and *faciei* caused by *Trichophyton tonsurans* that developed after starting the IL-17A/F inhibitor bimekizumab. Our case underlines how psoriatic patients, treated with IL-17 inhibitors, should be screened for cutaneous fungal infections before and during treatment, in order to exclude a concomitant infection or the risk of its exacerbation.

## 1 Introduction

Psoriasis is a complex, multifactorial immune-mediated disorder linked to the interleukin 17 (IL-17) signalling pathway activation [1]. The treatment of psoriasis has undergone a revolution with the advent of biologic therapies, including tumour necrosis factor (TNF)-α, IL-23 and IL-17 inhibitors. Among anti-IL-17 biologic agents, inhibitors, for the treatment of psoriasis are available ixekizumab and secukinumab, which are IL-17A inhibitors; brodalumab, an inhibitor of the IL-17A receptor; and bimekizumab, a monoclonal IgG1 antibody that inhibits both IL-17A and IL-17F [2]. All these monoclonal antibodies block the IL-17 signalling, improving psoriasis with remarkable efficacy and rapidity, more than TNF-α and IL-23 inhibitors [3]. The IL-17 cytokine family consists of six isoforms (IL-17A-IL-17F), whereas five members of the IL-17 receptor (IL-17R) family have been identified (IL-17RA-IL-17RE) [3]. The interleukin-17F and IL-17A contribute mainly to the pathobiology of plaque psoriasis and the simultaneous blockade of both cytokines may lead to more complete suppression of inflammation and superior clinical outcomes than blocking IL-17A alone [4]. In the BE SURE, BE VIVID and BE RADIANT phase III/IIIb trials, treatment with bimekizumab led to rapid and substantial clinical improvements in patients with moderate-to-severe plaque psoriasis [[3], [4], [5]]. In addition to improvements in clinical outcomes, the overall incidence of treatment-emergent adverse events (TEAEs) with bimekizumab was comparable with the other IL-17 inhibitors, except for an increased incidence of mild-to-moderate oral candidiasis, which did not lead to increased rates of treatment discontinuation [5,6]. As for psoriasis, IL-17-mediated diseases present dysregulation of this pathway, which plays a crucial role in host defence mechanisms against fungal pathogens, and iatrogenic modification of IL-17 axis could promote alterations of the microbial equilibrium in barrier tissues [7]. Rare congenital defects in the IL-17 pathway exemplify the relevance of IL-17 in protective immunity against the opportunistic fungal pathogen *C. albicans*. However, increasing evidence shows that IL-17 can also provide protection against various fungi other than *C. albicans*, including dermatophytes and other moulds [8,9]. Here, we report an unusual presentation of *tinea corporis* and *faciei* that developed after initiation of the IL-17 inhibitor bimekizumab.

## 2 Case presentation

**Fig. 1:**
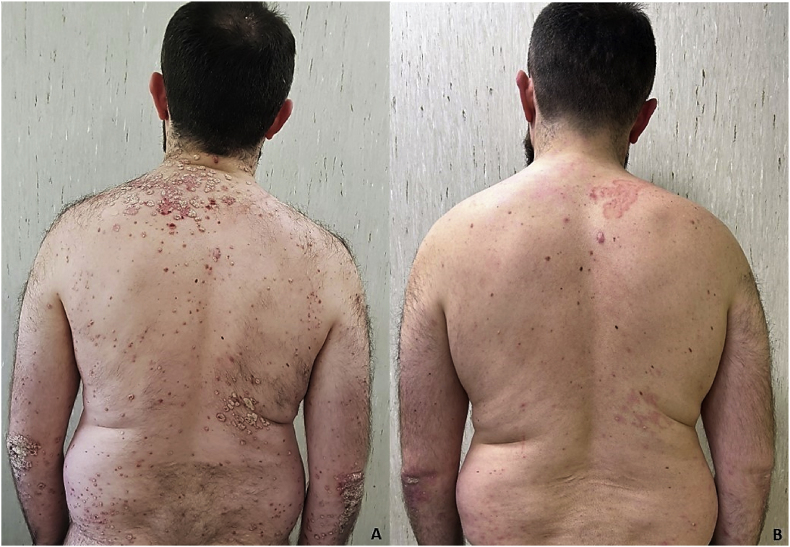
(A) Clinical evaluation at baseline revealed severe plaque psoriasis, located on back and arms with PASI 13. (B) After 4 weeks (Day +30) from baseline, the patient came to our attention with a complete resolution of cutaneous psoriasis, but a scaly, erythematous lesion was detected on the right shoulder with a clinical suspicion of *tinea corporis*.

We report a case of a 36-year-old Caucasian male with a 10-year history of plaque psoriasis. The patient was previously treated with topical medication, including methotrexate, cyclosporine and anti-TNF-α (Day −120) without improvement. At clinical evaluation (Day 0), moderate to severe plaque psoriasis was observed, with a Psoriasis Area and Severity Index (PASI) 13, including involvement of scalp and genital area (Fig. 1A). The patient was eligible to bimekizumab treatment and the first dose (160 mg ×2 subcutaneous) of the IL-17/A/F inhibitor was administered (Day 0).

**Fig. 2:**
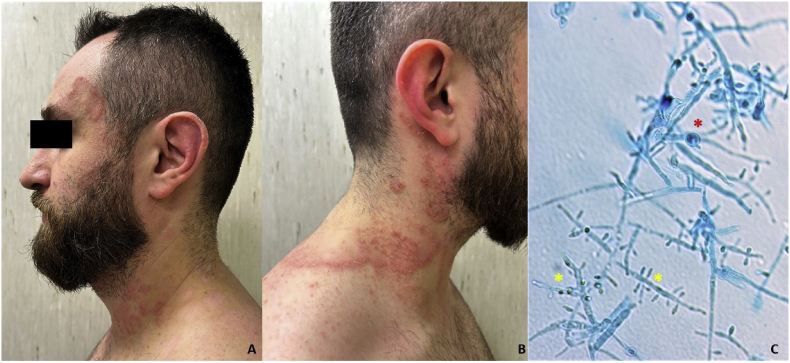
(A, B) Macroscopic examination revealed scaly, erythematous lesions on the left frontotemporal areas and on the neck with a clinical suspicion for tinea faciei and corporis (Day +30). (C) Microscopic examination showed broad, irregular, branched hyphae with septa. Several microconidia varying in size and shape (long clavate and broad pyriform) are borne at right angles to the hyphae (yellow asterisk). Swollen giant forms of microconidia and chlamydoconidia (red asterisks) are present. Microscopic features, supported by MALDI-TOF MS and PCR, are representative of *T. tonsurans* cutaneous infection. (For interpretation of the references to color in this figure legend, the reader is referred to the Web version of this article.)

After four weeks (Day +30) from the first administration of bimekizumab (160 mg ×2 subcutaneous), the patient presented a complete remission of plaque psoriasis on his back and harms (Fig. 1B,C), but at physical examination he presented an annular, erythematous scaly plaque with a serpiginous border on his neck, right shoulder and frontotemporal left area with clinical suspect of dermatophytosis (Fig. 2A,B). Skin scraping from the lesions was performed for a direct microscopic examination with KOH 40 %, highlighting the presence of fungal elements. Fungal cultures on Sabouraud Dextrose agar (SDA) (Difco Laboratories, Detroit, MI, USA), with and without cycloheximide, were performed and incubated at 25 °C and 37 °C for fungal isolation and identification. According to the national guidelines, he was prescribed terbinafine 250 mg, once daily, for three months, instructed to wash with an acid soap and apply ciclopirox 1 % cream twice daily for 1 month. During the same visit (Day +30) the patient was treated with the second dose of bimekizumab (160 mg ×2 subcutaneous), considering the mild extension of the fungal disease and the absence of other lesions or comorbities.

After 12 days (Day +40), powdery with a raised centre and radial grooves, yellow to brown colonies were detected on SDA supplemented with cycloheximide at 37 °C. Lactophenol blue mount made from a portion of the colony showed broad, irregular, branched hyphae with septa. Several microconidia, varying in size and shape (long clavate and broad pyriform), borne at right angles to the hyphae. Swollen giant forms of microconidia and chlamydoconidia were present (Fig. 2C), suspected of *Trichophyton* (*T.*) *tonsurans.* On the basis of microscopic features, Matrix-assisted laser desorption ionization time-of-flight mass spectrometry (MALDI-TOF MS) (Bruker Daltonics, MS, USA) method was performed and a score of 1.95 for *T. tonsurans* was obtained, confirming the microscopic examination. Mass spectrometry data analysis was performed by a MALDI Biotyper® (MBT) (Bruker Daltonics, Bremen, Germany), using the MALDI Biotyper software package (version 3.4) with the Filamentous Fungi Library 4.0 (Bruker Daltonics, Bremen, Germany). To definitively confirm the laboratory diagnosis of *T. tonsurans*, the DNA from a fungal culture was extracted and characterized by sequencing the Internal transcribed spacer (ITS) region (ITS1, 5.8S, and ITS2). The results from the sequencing analysis aligned with BLAST. NCBI (https://blast.ncbi.nlm.nih.gov/Blast.cgi (accessed on July 20, 2024)) confirmed the isolate as *T. tonsurans*. The results of the sequencing have been deposited in the GenBank with the following accession number: PQ423053.

**Fig. 3:**
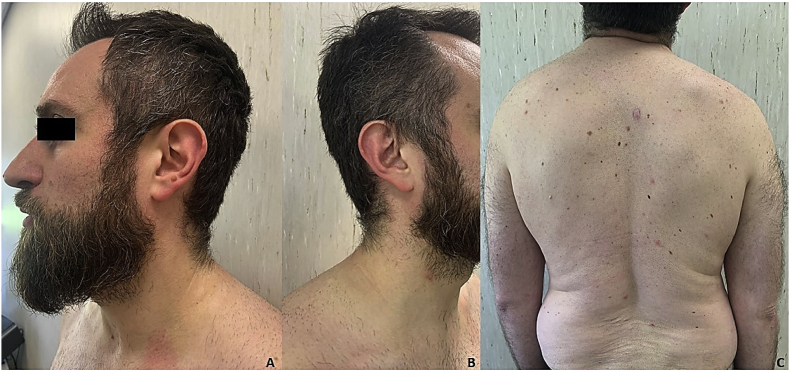
(A–C) Clinical evaluation at week 12 (day +60) showed the complete clinical remission of *tinea corporis* and *faciei* with concomitant PASI100.

The patient returned for follow-up 4 weeks later (Day +60) with clinical and mycological clearance of the lesions and a complete remission of plaque psoriasis, achieving PASI100: an improvement of 100 % with respect to the baseline PASI score (see Fig. 3). Neither adverse events with the concomitant use of systemic terbinafine and bimekizumab nor liver enzymes alterations have been reported.

Follow up visits every 8 weeks, until week 24 (Day +144) of bimekizumab administration, did not show other cutaneous fungal infections or relapse of *tinea corporis* and *faciei caused* by *T. tonsurans*.

## 3 Discussion

The field of IL-17 biology has gained much attention over the last decade owing to the pathogenic role of this cytokine in psoriasis and other autoinflammatory disorders and the successful implementation of IL-17-targeting therapies in patients suffering from these diseases [10]. Th-17 cells, producing IL-17A, IL-17F, and IL-22, were found to play a crucial role in antifungal immunity in barrier tissues [11]. Patients with defects of Th17 immunity pathway are unable to clear superficial *Candida* and dermatophytic infections. In fact, it has been demonstrated that chronic mucocutaneous candidiasis (CMC) occurs in patients with autosomal recessive IL‐17RA deficiency and autosomal dominant IL‐17F deficiency [12]. Moreover, the gain-of-function mutations in signal transducer and activator of transcription (STAT) 1 have been reported as key factors in conferring an increased risk for the development of CMC. This is due to an impairment of STAT3 activity in a STAT1-dependent manner, resulting in a reduction in the development of T cells producing IL-17A, IL-17F and IL-22 [12]. Although the risk of candidiasis undertreatment with IL-17 inhibitors is described, scanty data are available regarding dermatophytic infections. Quach et colleagues [13] reported 2 cases of perianal dermatophytic infections and 4 cases of *tinea pedis* during secukinumab treatment for plaque psoriasis. Diruggero et al. [14] described one case of *tinea incognito* undertreatment with brodalumab. In addition, Strober et al. [15] reported that *tinea pedis,* under ixekizumab therapy, occurred in 1.3/100 patient-year (in 7 trials evaluating ixekizumab, for a total 6479.8 patient-year). McInnes et *al*. [16] reported a case of *tinea versicolor* due to *Malassezia* spp. in a patient with psoriatic arthritis. However, no data on dermatophytic infections with bimekizumab have been published so far. Since IL-17 stimulates the production of keratinocyte-produced antimicrobial peptides such as human beta-defensin-2 and catelicidin (LL-37), and also promotes neutrophil recruitment, the inhibition of IL-17A, IL-17 and the heterodimer IL-17A/F may reduce mucocutaneous innate immunity and, thus, predispose to superficial dermatophytosis. Moreover, Herein, we report a case of *tinea corporis* and *faciei* arising 4 weeks after the first dose of bimekizumab. Given the rapid and widespread onset of the disease, it is possible that the subject had a non-clinically visible *tinea corporis* behind psoriasis, and treatment with bimekizumab led to a complete remission of psoriatic disease but made clinically evident the fungal infection. Another hypothesis is that the patient was an asymptomatic carrier of *T. tonsurans* [17] and the systemic therapy with IL-17 inhibitor could cause an impairment in the microbial equilibrium in barrier tissues as skin, promoting the infection. Although the incidence of fungal infections was found to be increased during anti-IL-17 therapy, patients undergoing such treatment should be screened and monitored for mycotic infections. Saunte et al. [18] proposed a screening algorithm for *Candida* infections in patients who receive anti-IL-17 drugs, including clinical signs and microbiological assessment. On the other hand, a previous study by our group [9] suggests that the evaluation of the serum levels of All-Trans Retinoic Acid -ATRA, the active form of vitamin A-should be considered as a predictive biomarker for the development of mycoses among psoriatic patients treated with IL-17 inhibitors. Our case emphasizes the importance of screening psoriatic patients for fungal infections before and after the treatment with anti-IL-17 biologics in order to exclude a concomitant undiagnosed superficial mycosis or the risk of its exacerbation.

## CRediT authorship contribution statement

**Terenzio Cosio:** Conceptualization, Methodology, Software, Writing – original draft. **Ruslana Gaeta Shumak:** Investigation. **Cristiana Borselli:** Investigation. **Fabio Artosi:** Investigation. **Roberta Gaziano:** Supervision, Writing – original draft. **Elena Campione:** Supervision, Visualization.

## 4 Ethical form

The authors confirm that this material is original and has not been published in whole or in part elsewhere; that the manuscript is not currently being considered for publication in another journal; and that all authors have been personally and actively involved in substantive work leading to the manuscript and will hold themselves jointly and individually responsible for its content.

## Declaration of competing interest

There are none.
